# Notch pathway activation enhances cardiosphere in vitro expansion

**DOI:** 10.1111/jcmm.13832

**Published:** 2018-08-23

**Authors:** Ilaria Secco, Lucio Barile, Consuelo Torrini, Lorena Zentilin, Giuseppe Vassalli, Mauro Giacca, Chiara Collesi

**Affiliations:** ^1^ Molecular Medicine Laboratory International Centre for Genetic Engineering and Biotechnology (ICGEB) Trieste Italy; ^2^ Fondazione Cardiocentro Ticino and Swiss Institute for Regenerative Medicine Lugano Switzerland; ^3^ Department of Medical Surgical and Health Sciences University of Trieste Trieste Italy

**Keywords:** Notch, Adeno‐associated virus (AAV), Micro‐RNAs, Cardiac Progenitors

## Abstract

Cardiospheres (CSps) are self‐assembling clusters of a heterogeneous population of poorly differentiated cells outgrowing from in vitro cultured cardiac explants. Scanty information is available on the molecular pathways regulating CSp growth and their differentiation potential towards cardiac and vascular lineages. Here we report that Notch1 stimulates a massive increase in both CSp number and size, inducing a peculiar gene expression programme leading to a cardiovascular molecular signature. These effects were further enhanced using Adeno‐Associated Virus (AAV)‐based gene transfer of activated Notch1‐intracellular domain (N1‐ICD) or soluble‐Jagged1 (sJ1) ligand to CSp‐forming cells. A peculiar effect was exploited by selected pro‐proliferating miRNAs: hsa‐miR‐590‐3p induced a cardiovascular gene expression programme, while hsa‐miR‐199a‐3p acted as the most potent stimulus for the activation of the Notch pathway, thus showing that, unlike in adult cardiomyocytes, these miRNAs involve Notch signalling activation in CSps. Our results identify Notch1 as a crucial regulator of CSp growth and differentiation along the vascular lineage, raising the attracting possibility that forced activation of this pathway might be exploited to promote in vitro CSp expansion as a tool for toxicology screening and cell‐free therapeutic strategies.

## 1 INTRODUCTION

The heart of adult mammals has a limited capacity of replacing damaged tissue after injury. Understanding the molecular mechanisms of cardiomyocyte proliferation during development, as well as of the incapacity of mature cardiomyocytes to divide in adult mammals, is a pre‐requisite for the development of innovative approaches to prevent loss of functional myocardium after injury.

Experimental evidence over the past few years has suggested that cardiac regeneration might be achieved by directly stimulating the proliferation of committed cardiac progenitors, or even of fully differentiated cardiomyocytes.^1^, ^2^ However, cell cycling is very low in adult cardiomyocytes and myocardial repair after damage usually involves scarring and fibrosis, which frequently leads to heart failure. Thus, the identification of strategies capable of inducing cardiac tissue regeneration by sustaining cardiomyocyte generation would be of paramount importance. In recent years, multiple laboratories have independently reported on the identification of resident cardiac progenitor cells (CPCs) on the basis of the expression of cell‐surface markers such as stem cell antigen‐1 (Sca‐1) or c‐Kit, and co‐expression of early cardiac‐specific transcription factors such as Gata4 or Nkx2.5 (for a review see^3^). These cells have been used in several cell therapy approaches aiming at promoting regeneration of damaged hearts (for a review see^4^, ^5^). The functional benefits of transplanted CPCs have been mainly ascribed to indirect paracrine mechanisms mediated by secreted factors, as opposed to direct generation of new cardiomyocytes^6^, ^7^).

In the context of cardiac progenitor cell proliferation and expansion, a crucial role is played by the Notch signalling pathway, a well‐conserved cell‐to‐cell communication system that critically regulates embryonic heart development.^8^, ^9^ Most specifically, Notch regulates cardiomyocyte proliferation during development and in the early postnatal life. In particular, we and others have shown that the Notch pathway promotes the proliferation of committed precursor cells and immature cardiomyocytes^10^, ^11^ and is essential for the maintenance of the heart structural and functional integrity after damage in mammals as well as in lower vertebrates,^12^, ^13^ in which it has been identified as a central mechanism in heart regeneration.^14^, ^15^ In CPCs, Notch activation prevents premature cardiomyogenic differentiation, while promoting proliferation in transient amplifying populations;^16^, ^17^ conversely, inhibition of the Notch pathway is required to promote cardiac mesoderm differentiation in embryonic stem cells.^18^ Committed undifferentiated progenitor cells can be clonally expanded from myocardial biopsy specimens and cultured in vitro as tridimensional spheroids.^19^, ^20^ Cardiospheres (CSps) are cardiac‐derived multicellular clusters, considered a reliable in vitro model of the cardiac microenvironment, hosting a reservoir of both immature and committed progenitors towards the three major cardiac cell lineages: cardiomyocytes, endothelial cells (ECs) and smooth muscle cells (SMCs).^21^ CSps transplantation into infarcted hearts results in enhanced in vivo cell survival and cardioprotection.^22^ Recent data have shown that activation of the Notch signalling pathway promotes cell differentiation along the vascular smooth muscle lineage in CSp‐derived cells and is sensitive to oxygen tension in the tridimensional cellular array.^23^

In this work, we aimed at dissecting the specific role of Notch1 signalling in CSps. We report that Notch1 stimulation by soluble Jagged‐1 triggers a massive increase in both number and size of in vitro cultured CSps. This effect was paralleled by a significant increase in vascular developmental markers such as Flt‐1, Kdr and CD31 and the adoption of a specific gene expression signature. Conversely, the γ‐secretase inhibitor, DAPT, which impairs Notch1 activation, effectively blunted in vitro growth of sJ1‐stimulated CSps, as well as modulated their gene expression pattern. These effects were further enhanced using Adeno‐associated virus (AAV)‐based gene transfer of activated Notch1‐intracellular domain (N1‐ICD) or the soluble‐Jagged1 ligand (sJ1) to CSp‐forming cells. These viral vectors possess the exquisite capacity to transduce cardiac‐derived cells at very high efficiency and drive sustained transgene expression.^24^, ^25^

Finally, a whole‐genome, synthetic miRNA screening previously conducted in our laboratory has identified a pool of human miRNAs capable of inducing significant proliferation in rodents cardiomyocytes.^26^ Notably, the molecular mechanisms by which these miRNAs function in adult cardiomyocytes do not involve reactivation of the Notch pathway.^24^ We found that, among all tested miRNAs, hsa‐miR‐590‐3p was the most powerful inducer of a cardiovascular gene expression programme in CSps, leading to a significant increase in both early (Gata4, Nkx2.5) and late cardiac markers (Myh6), as well as of vascular markers (CD31).

Collectively, our results identify Notch1 as a crucial regulator of CSps growth and differentiation along the vascular lineage, raising the possibility that forced activation of this signalling pathway might be exploited to promote in vitro CSps growth not only for therapeutic purposes but also as a tool for evaluating novel cell‐free therapeutic strategies (for example, those based on the delivery of exosomes or miRNAs).

## 2 METHODS

### 2.1 Animals

C57bl/6 and CD1 mice were purchased from Charles River Laboratories Italia Srl and maintained under controlled environmental conditions. Animals were housed and handled according to institutional guidelines in compliance with national and international laws and policies. Experimental procedures were approved by the ICGEB Animal Welfare Board and Ethical Committee, with full respect to the EU Directive 2010/63/EU for animal experimentation.

### 2.2 Culture of rodent cardiospheres

CSps were obtained and cultured as previously described.^27^ Briefly, isolated myocardial tissue was cut into 1‐2 mm^3^ pieces, washed in PBS and digested 3 times for 5 minutes at 37°C with 0.2% trypsin (Invitrogen, Carlsbad, Ca, USA). Following trypsin inactivation in complete explant medium (CEM) (Iscove's Modified Dulbecco's Medium [IMDM] supplemented with 20% fetal bovine serum, 100 U/mL penicillin G, 100 μg/mL streptomycin, 2 mmol/L L‐glutamine and 0.1 mmol/L 2‐mercaptoethanol), tissue fragments were cultured as explants in CEM at 37°C and 5% CO_2_. After approximately 2 weeks, a layer of fibroblast‐like cells was generated from adherent explants over which small, phase‐bright cells migrated. These phase‐bright cells were collected by pooling 2 washes with Ca^2+^‐Mg^2+^‐free PBS, 1 wash with 0.53 mmol/L EDTA (Versene, Invitrogen) (2 minutes), and 1 wash with 0.5 g/L trypsin and 0.53 mmol/L EDTA (Invitrogen) (2‐3 minutes) at room temperature under visual control. The cells obtained (from 10^4^ to 4 x 10^5^ cells/explant) were seeded at 1‐2 x 10^4^ cells/mL in poly‐D‐lysine‐coated multiwell plates (BD Biosciences, Franklin Lakes, NJ, USA) in CSp‐growing medium (CGM‐35% complete IMDM/65% DMEM‐Ham F‐12 mix containing 2% B27, 0.1 mmol/L 2‐mercaptoethanol, 10 ng/mL epidermal growth factor,^28^ 20 ng/mL basic fibroblast growth factor [bFGF], 40 nmol/L cardiotrophin‐1, 40 nmol/L thrombin, antibiotics and L‐Glu, as in CEM). Isolation of the CSp‐forming cells was performed from the same explant at least 4 times at 6‐10 days intervals.

### 2.3 miRNA transfection

MicroRNAs were obtained from Dharmacon, Thermo Scientific and transfected into CSp‐forming cells contextually to plating on poly‐K coated dishes using a standard reverse transfection protocol.^26^ Briefly, the transfection reagent (Lipofectamine RNAiMAX, Life Technologies, Carlsbad, Ca, USA) was diluted in OPTIMEM (Life Technologies) and added to the miRNAs (at a final concentration of 25 nmol/L each nucleic acid), arrayed on 96‐well plates. Thirty minutes later, 1 x 10^4^ cells were seeded per well. Twenty‐four hours after transfection, culture medium was replaced by fresh medium. Cells were fixed 3, 5 and 7 days after plating and processed for further analyses. Experiments were performed in quadruplicate.

### 2.4 RNA isolation and quantitative real‐time PCR

Total mRNA was purified from CSps at days 3, 5 and 7 of culture. mRNA (1 μg) was reverse‐transcribed using MLV‐RT (Invitrogen) with random hexamers (10 μmol/L) in a 20‐μL reaction, following the manufacturer's instruction. mRNA levels for Notch1, Notch2, Notch3, Notch4, Jagged1, Jegged2, Delta‐like1, Delta‐like3, Delta‐like4, Hes1, Hey1, Sca‐1, CD117, CD31, Flt1, Kdr, Nkx2.5, Gata4, Myh6, Neurl1a, Dner and HPRT genes (TaqMan assays listed in Table 1) were quantified by real‐time PCR, performed with a standard probe protocol (from 60 to 95°C), according to manufacturer's instruction. The housekeeping gene HPRT was used for normalization with predesigned TaqMan assay (Applied Biosystems, Foster City, Ca, USA) and iQ Supermix (Biorad, Hercules, Ca, USA). mRNA levels for N1ICD and sJ1 transgenes were quantified by quantitative real‐time PCR (primer sequences listed in Table 2) and GoTaq qPCR Mater Mix (Promega, Madison, Wi, USA).

**Table 1:** TaqMan real‐time PCR assays used to analyse gene expression levels

Notch1	Mm00627185_m1
Jagged1	Mm00496902_m1
Notch2	Mm00803077_m1
Delta‐like1	Mm01279269_m1
Hes1	Mm01342805_m1
Hey1	Mm00468865_m1
Hey2	Mm00469280_m1
CD117	Mm00445212_m1
Sca‐1	Mm00485928_m1
CD31	Mm01242584_m1
Flt1	Mm00438980_m1
Kdr	Mm01222421_m1
NKx2.5	Mm01309813_s1
Gata4	Mm00484689_m1
Myh6	Mm00440359_m1
Notch3	Mm01345646_m1
Notch4	Mm00440525_m1
Jag2	Mm01325629_m1
Dll3	Mm00432854_m1
Dll4	Mm00444619_m1
Dner	Mm00548872_m1
Neurl1a	Mm00480473_g1

**Table 2:** Primers used to analyse gene expression levels

sJ1	Forward	ATTTCTGCTGAAGATATAGCCC
	Reverse	CTCCATTTCATTCAAGTCCTC
N1ICD	Forward	AGCAAGGAAGCTAAGGACC
	Reverse	CTCCATTTCATTCAAGTCCTC
HPRT	Forward	CAGTCAACGGGGGACATAAA
	Reverse	GGGCTGTACTGCTTGACCAA

### 2.5 Immunocytochemical staining

CSps were fixed with 4% paraformaldehyde for 10 minutes, permeabilized with 0.1% Triton X‐100 in phosphate buffered saline (PBS) for 10 minutes, followed by 1 hour blocking in 2% BSA (Roche, Basel, Switzerland) in PBS. BrdU staining of CSps pulsed 8 hours prior fixation with 10‐μmol/L BrdU, was performed according to the manufacturer's instructions (BD Biosciences). Cells were then stained overnight at 4°C with the following primary antibodies diluted in blocking solution: rabbit polyclonal anti‐Notch1 (C20R‐Santa Cruz, Dallas, Texas, USA), 1:100; rabbit polyclonal anti‐Notch2 (C20R‐Santa Cruz, Dallas, Texas, USA), 1:100; rabbit polyclonal anti‐Val1744 (Cell Signaling, Danvers, Massachusetts, USA), 1:100; rabbit polyclonal anti‐Jagged1 (Santa Cruz, Dallas, Texas, USA), 1:100; goat polyclonal anti‐Dll1 (C20R‐Santa Cruz, Dallas, Texas, USA), 1:50; rabbit polyclonal anti‐Hes1 (Chemicon, Temecula, Ca, USA), 1:100; rat monoclonal anti‐BrdU (BU1/75) 1:100 (AbCam, Cambridge, UK). Cells were washed with PBS and incubated for 1 hour with the respective secondary antibodies (goat anti‐mouse conjugated to Alexa Fluor 488; goat anti‐rabbit conjugated to Alexa Fluor 594; goat anti‐rat conjugated to Alexa Fluor 594). All washes were in PBS 0.2% Tween 20. When indicated, cells were further processed using the Click‐IT EdU 555 Imaging kit to reveal EdU incorporation, according to the manufacturer's instructions, and stained with Hoechst 33342 (Life Technologies).

### 2.6 Image acquisition and analysis

Images were acquired at room temperature with a DMLB upright fluorescence microscope (Leica, Wetzlar, Germany) equipped with a charge‐coupled device camera (CoolSNAP CF; Roper Scientific, Trenton, NJ, USA) using MetaView 4.6 quantitative analysis software (MDS Analytical Technologies, Sunnyvale, Ca, USA). For image acquisition, the following objectives were used: HCX PL Fluotar 100x/1.30 NA, HCX PL apocromatic 63x/1.32‐0.6 NA, HCX PL Fluotar 40x/0.75 NA, HCX PL N‐Plan 20x/0.40 NA and HCX PL N‐Plan 10x/0.25 NA (all from Leica). Within each experiment, instrument settings were kept constant.

### 2.7 AAV vector production and transduction

The AAV vectors used in this study were generated by the AAV Vector Unit (AVU) at ICGEB Trieste (http://www.icgeb.org/avu-core-facility.html) according to established procedures. All vectors were based on the AAV2 genome and transgene expression was driven by the cytomegalovirus immediate/early promoter. CSp‐forming cells were transduced with AAV6‐Control, AAV6‐sJ1, AAV6‐N1ICD or AAV6‐GFP contextually to plating on poly‐lysine coated dishes, at a multiplicity of infection (m.o.i.) of 1 x 10^5 ^vg/cell.

### 2.8 Statistical analysis

All data are presented as mean ± standard error of the mean (SEM). Statistical analysis was carried out with Prism Software (GraphPad) using one‐way ANOVA followed by Bonferroni's post hoc test for comparisons of 3 or more groups.

## 3 RESULTS

### 3.1 CSps as an in vitro culture model of heart cell precursors

**Figure 1:**
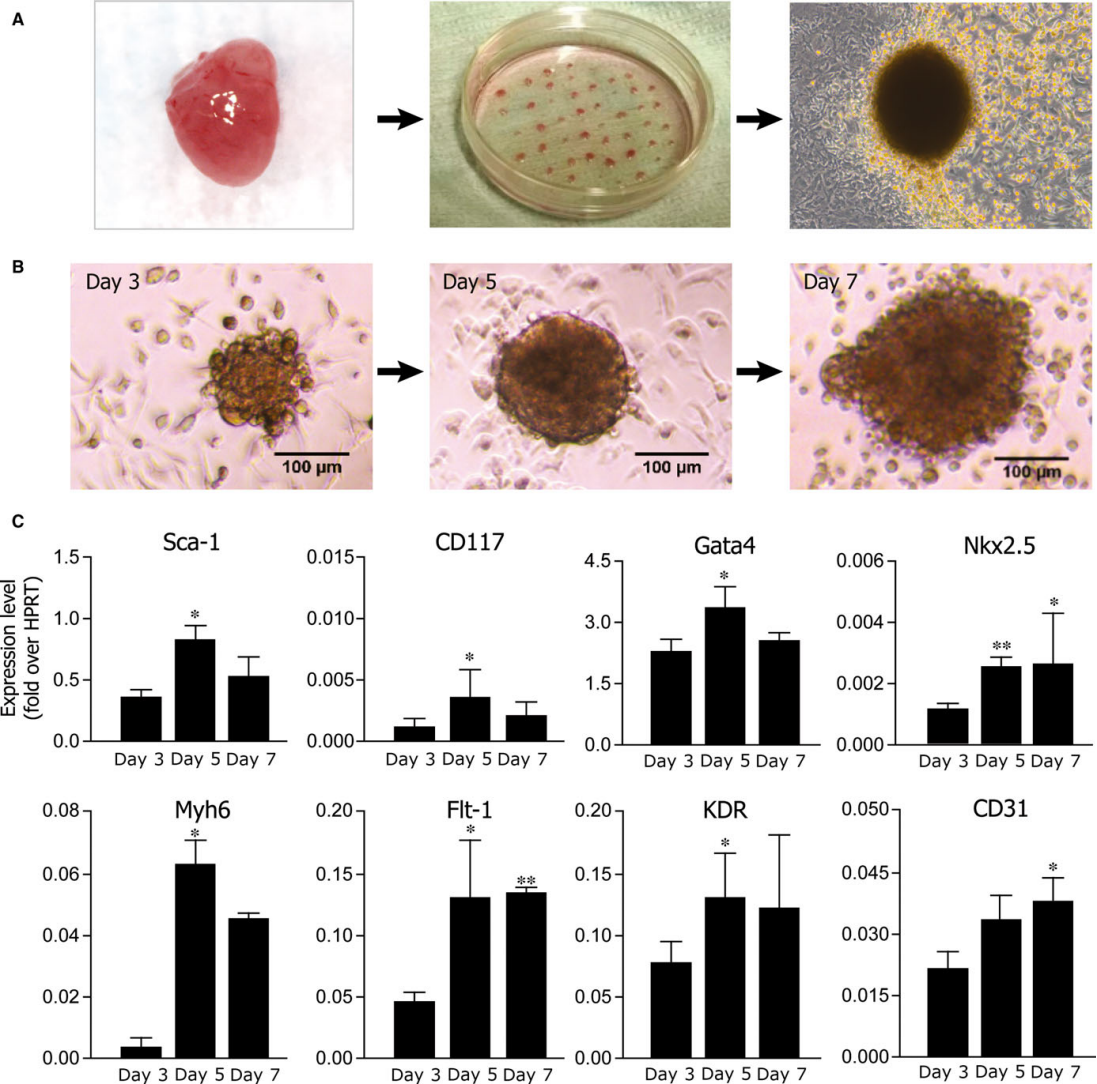
Derivation and characterization of cardiospheres. A, Schematic diagram showing derivation and culture of cardiospheres; B, Representative bright field images of primary cardiospheres at 3, 5 and 7 days after plating; scale bars: 100 μm. C, Gene expression profile of cultured cardiospheres at days 3, 5 and 7 after plating. Shown are the mean ± SEM of at least 3 independent experiments. *P <* *0.05; **P <* *0.01

Notch regulates cardiomyocyte proliferation during development and in the early postnatal life. We and others have previously shown that the Notch pathway promotes the proliferation of committed precursors and immature cardiomyocytes.^10^, ^16^, ^29^, ^30^ We thus investigated the specific role of Notch signalling in the pool of cardiac‐resident precursor cells in the murine CSp model^19^, ^27^ (Figure 1A). Analyses were performed at three time points: 3 days after cell plating (the time point of earliest CSp formation), 5 days after plating (the time point of fully CSp assembly) and 7 days after plating (the time point at which most CSps stop increasing in number and size; Figure 1B). To characterize the cell differentiation programme during CSp formation, we analysed expression of genes related to stemness (CD117 and Sca‐1), cardiac commitment (Nkx2.5, Gata4, Myh6) and endothelial specification (CD31, Flt1 and KDR). The real‐time PCR results shown in Figure 1C led us to identify three main cell populations.

1. The bell‐shaped expression profile of both CD117 and Sca‐1 demonstrated the persistence, during the CSp aggregation process, of a pool of Sca1+ progenitors, which decreased after one week of culture.
2. The expression of the endothelial marker CD31, together with VEGF receptors 1 and 2 (Flt1 and KDR, respectively) demonstrated the presence of cells with vasculo‐endothelial commitment potential.
3. Nkx2.5, Gata4 and Mhy6 expression identified a third pool of cells characterized by cardiac differentiation markers.

Collectively, these evidences were consistent with the notion that CSps actually represent a valuable in vitro model of early cardiac development.

### 3.2 Notch signalling components in CSps

**Figure 2:**
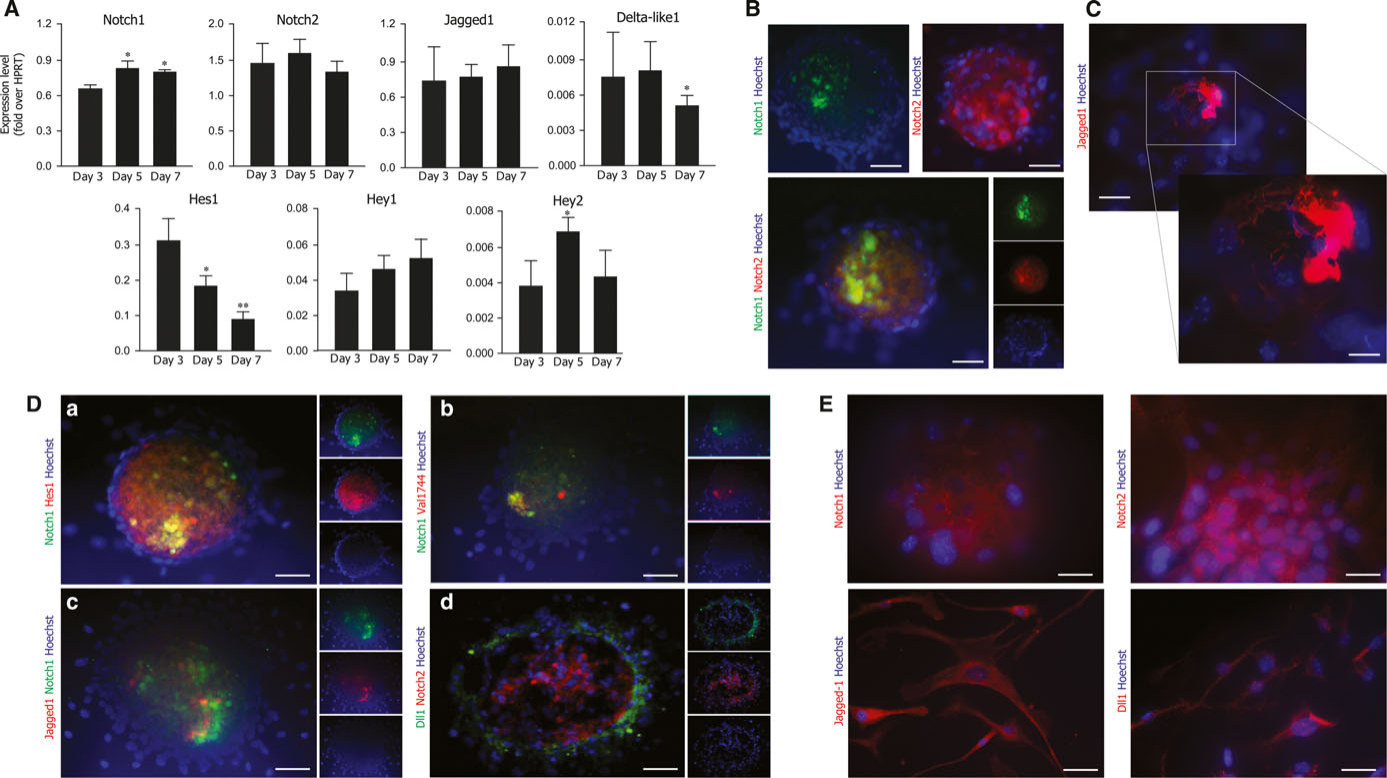
Expression of the Notch pathway components in cardiospheres. A, Gene expression analysis of Notch pathway components in cultured cardiospheres analysed at days 3, 5 and 7 after plating. Shown are the means ± SEM of at least 3 independent experiments. **P < 0.01; *P < 0.05. B, Immunolocalization of Notch receptors in cardiospheres after 5 days of culture. Representative images of cardiospheres stained for Notch1 (green), Notch2 (red); nuclei are visualized in blue by Hoechst. Bars: 40 μm. C, Same as in panel B for the Jagged1 ligand. Jagged1 (red); nuclei are visualized in blue by Hoechst. Bars: 40 μm. D, Same as in panel B for (a) Notch1 (green) and Hes1 (red); (b) Notch1 (green) and N1‐ICD (Val 1744‐red); (c) Jagged1 (red) and Notch1 (green); (d) Dll1 (green) and Notch2 (red). Nuclei are visualized in blue by Hoechst. Bars: 40 μm. E, Immunolocalization of Notch receptors and ligands in CDCs. Representative images of cardiospheres stained for Notch1, Notch2, Jagged1 and Dll‐1 (red); nuclei are visualized in blue by Hoechst. Bars: 40 μm

To investigate a possible role of the Notch pathway during CSp formation, we analysed the expression levels of the Notch receptors (Notch1, Notch2), their ligands (Jagged1, Delta‐like1) and their target genes (Hes1, Hey1, Hey2) at 3, 5 and 7 days after plating (a panel of all the members of the receptor and ligand family are shown in Figure S1). Figure 2A shows that both Notch1 and 2 receptors as well as the Jagged1 and Dll‐1 ligands were expressed with a bell‐shaped pattern during CSp formation. Hey2 showed an expression profile superimposable to that of Notch1 and 2. Hes1 expression declined during CSp formation, while Hey1 showed the opposite pattern. These data suggest that Hes1 and Hey proteins may play different roles in the CSp differentiation programme. Hes1 is the main Notch effector during embryonic heart development and morphogenesis, and Hey2 plays an important role in the regulation the cardiovascular development. Conversely, Hey1 exerts its role of transcriptional activator during vasculogenesis, angiogenesis and blood vessel development.^31^ Therefore, it is intriguing that the expression of Hey1 increases during CSp formation, in parallel with the observed increase in the expression of VEGFR genes (Figure 1C).

One major constrain of real‐time PCR analysis is that the cell‐type expressing a given gene is not precisely identified. To address this issue, we performed immunostaining experiments on CSps after 5 days of culture, a time point at which sphere formation is complete and all our genes of interest are expressed at significant levels. Notch2 showed a diffuse localization pattern within the CSp, while immmunodetection of both Notch1 and its ligand Jagged1 revealed a more patchy distribution; Delta‐like1 was localized mainly in the outer layer of CSps (Figure 2B, C, D panel c and d). The expression of both Notch1 and Notch2 receptors, as well as that of Jagged1 and Dll‐1, was also observed in substrate‐adherent CSp‐forming cells (Figure 2E).

We also investigated the subcellular localization of Hes1 and N1‐ICD, two key players of the activated Notch pathway. Hes1 showed a diffuse staining in the inner part of CSps, while activated N1‐ICD (detected by an antibody raised against the first amino acids of its cleaved intracellular domain) exhibited significant co‐localization with Notch1 (Figure 2D panels a and b). These results suggested an active role of Notch signalling in CSp formation and maturation.

### 3.3 Stimulation of the Notch pathway enhances CSp growth

In several models of stem cell in vitro culture, Notch expression co‐localizes with pools of proliferating cells.^18^, ^32^, ^33^ To directly assess this issue, we cultured CSps in the presence of the conditioned medium from NIH‐3T3 cells secreting a soluble form of Jagged1 (sJ1; 34). In a mirror experiment, we treated cells with the γ‐secretase inhibitor, DAPT, to investigate whether blocking Notch activation impairs CSp formation and growth.

The effect of sJ1 has been extensively characterized in our laboratory.^10^, ^24^, ^35^ Current evidence suggests that sJ1 may spontaneously cluster on the surface of the target cells by virtue of its interactions with various proteins of the extracellular matrix and cell surface, as described for other soluble growth factors (for a review see^36^, ^37^, ^38^). sJ1 therefore could activate Notch signalling acting as a soluble cytokine.

**Figure 3:**
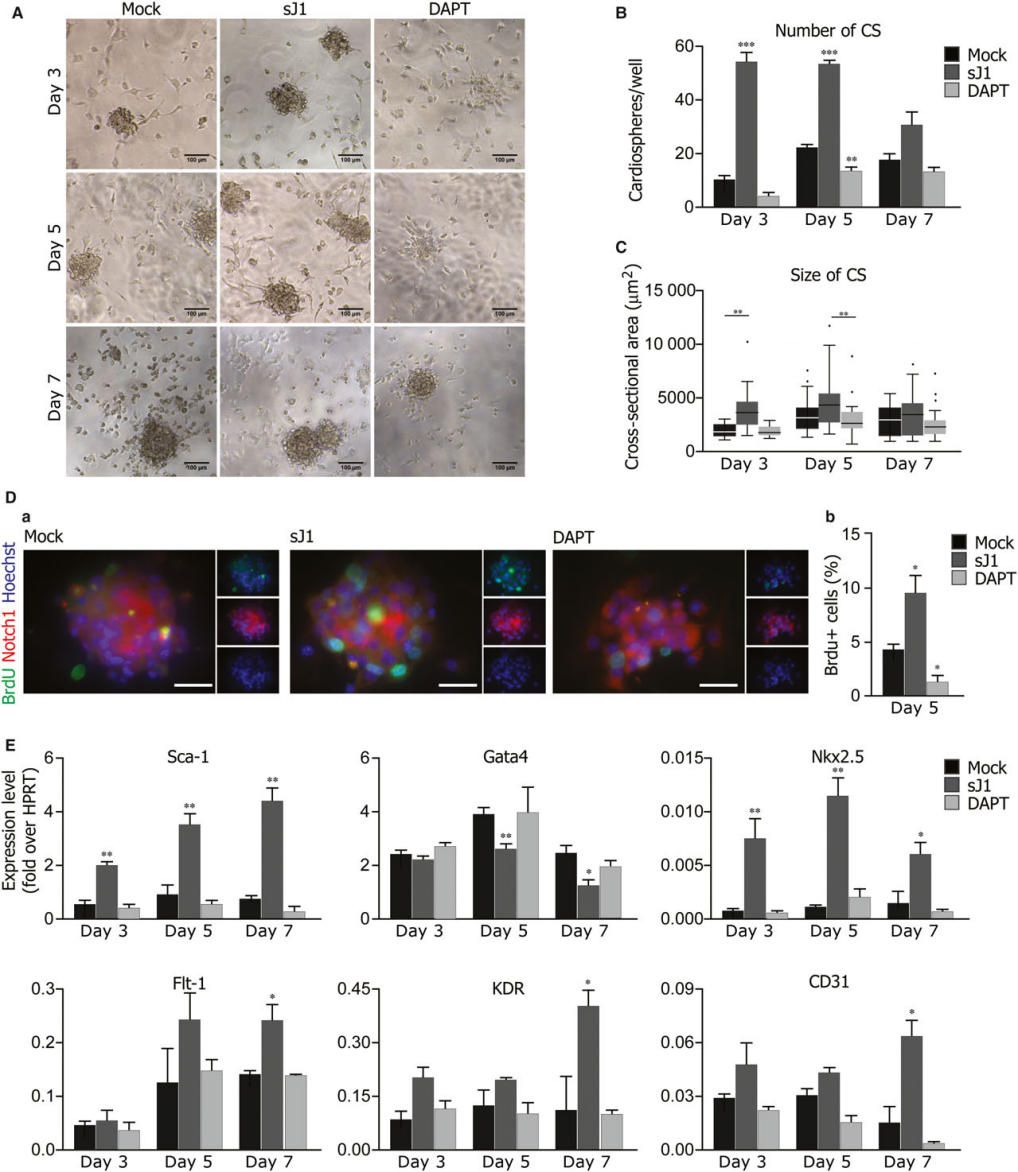
Stimulation of the Notch pathway enhances cardiosphere growth and induces a cardiovascular differentiation programme. Cardiosphere‐forming cells were treated either with the soluble form of Jagged1 (sJ1) or with DAPT for 24 h before the analysis after 3, 5 and 7 days of in vitro culture A, Representative bright field images of cardiospheres, treated either with 20x sJ1 or DAPT, after 3, 5 and 7 days of in vitro culture. Scale bar: 100 μm. B, Quantification of the number cardiospheres treated either with 20x sJ1 or DAPT, after 3, 5 and 7 days of in vitro culture. Shown are mean ± SEM of at least 3 experimental replicates ***P < 0.001; **P < 0.01. C, Distribution of the cross‐sectional areas of the cardiospheres, treated either with 20x sJ1 or DAPT, after 3, 5 and 7 days of in vitro culture. Shown are Tukey boxplots, **P < 0.01. D, (a) Representative images of BrdU+ cells in cardiospheres after 5 days of in vitro culture in the presence of 20X sJ1 or DAPT. BrdU (green), Notch1 (red), Nuclei (blue). Scale bar 40 μm. (b) Quantification of BrdU+ cells upon sJ1 or DAPT treatment. Shown are mean ± SEM of at least 3 experimental replicates. **P < 0.01: *P < 0.05. E, Transcription levels of indicated genes in cardiospheres analysed at day 3, 5 and 7 of in vitro culture, in the presence of either 20x sJ1 or DAPT. Data are expressed to cellular HPRT mRNA levels. Shown are the mean ± SEM of at least 3 independent experiments. **P < 0.01: *P < 0.05

Exposure to sJ1 at 3 or 5 days after cell plating significantly boosted the aggregation capacity of CSp‐forming cells; however, this effect was blunted when sJ1 was added to cells 7 days after plating. On the other hand, DAPT treatment strongly reduced the number of CSps formed at any time point (Figure 3A, B). Notch pathway activation showed a remarkable effect not only on the total number of CSps, but also on their size. sJ1 treatment significantly increased the cross‐sectional area of CSps, whereas DAPT significantly decreased their overall diameter at day 5 of culture (Figure 3C). The number of BrdU‐positive cells in CSps was increased in sJ1‐stimulated conditions after 5 days of culture, which was paralleled by increased expression of the Notch1 receptor (Figure 3D, panel a and b for quantification).

Collectively, these results strongly support the conclusion that Notch1 activation powerfully stimulates the proliferation and expansion of CSp‐forming cells at early times of culture; however, this effect progressively decreases during CSp differentiation.

### 3.4 Stimulation of the Notch pathway promotes a cardiovascular gene expression programme in growing CSps

To further investigate the signalling network downstream Notch activation, which enhances CSp growth, we analysed gene expression profiles of CSps after 3, 5 and 7 days of culture in the presence of either sJ1 or DAPT. sJ1 triggered a net increase in the expression of both Notch receptors (Notch1 and ‐2) and ligands (Jagged1 and Delta‐like1), consistent with the existence of a positive feed‐back loop,^13^, ^39^ whereas γ‐secretase inhibition reduced their overall levels.

We also assessed mRNA expression levels for endogenous Notch targets. Gene expression of Hes1 and, to a lesser extent, Hey2 (two major transducers of Notch signalling during heart development) paralleled the increase in CSp growth (Figure S2). We next analysed the expression of genes related to stemness, blood vessel or heart development. Figure 3E shows that sJ1 stimulation markedly increased expression levels of the genes involved in angiogenesis, such as CD31 and VEGF receptors 1 and 2. In addition, sJ1 stimulation was associated with increased expression of Nkx2.5, the master gene for cardiac development, but decreased Gata4 expression, in agreement with published data.^16^, ^40^ Unexpectedly, Sca1 expression increased after sJ1 stimulation.

These results support the notion that CSp differentiation requires a fine tuning of the Notch pathway. As demonstrated under different experimental conditions, Notch signalling must be turned off in order to trigger committed progenitor cell differentiation into a fully cardiac phenotype.^8^, ^41^ On the other hand, hypoxia associated with the tridimensional array of cells typical of CSps greatly favours the activation of the Notch pathway and the expansion of the pool of proliferating cells responsible for the sustained growth in vitro.^18^

### 3.5 Activation of the Notch pathway by AAV‐mediated gene transfer promotes CSp growth

Given the effect of the exogenous stimulation of the Notch pathway by sJ1 on CSps number and size, we asked whether the forced and sustained activation of the Notch pathway using a gene transfer approach would enhance these effects. We took advantage of the ability of AAV‐vectors, serotype 6 (AAV6), to transduce cardiac cells at high efficiency (Figure 3A; 25). We used two AAV6 vectors, one coding for the constitutively active Notch1 intracellular domain (AAV6‐N1ICD; 10) and the other coding for the soluble form of the ligand Jagged1 (AAV6‐sJ1; 24), which activates Notch signalling acting as a soluble cytokine.^10^, ^42^ CSp‐forming cells were transduced contextually to their plating (m.o.i = 1 x 10^5^ vg per cell) with either vector. Analyses were performed at 3, 5 and 5 days after plating. Sustained transgene expression was demonstrated with both vectors (Figure S3B).

**Figure 4:**
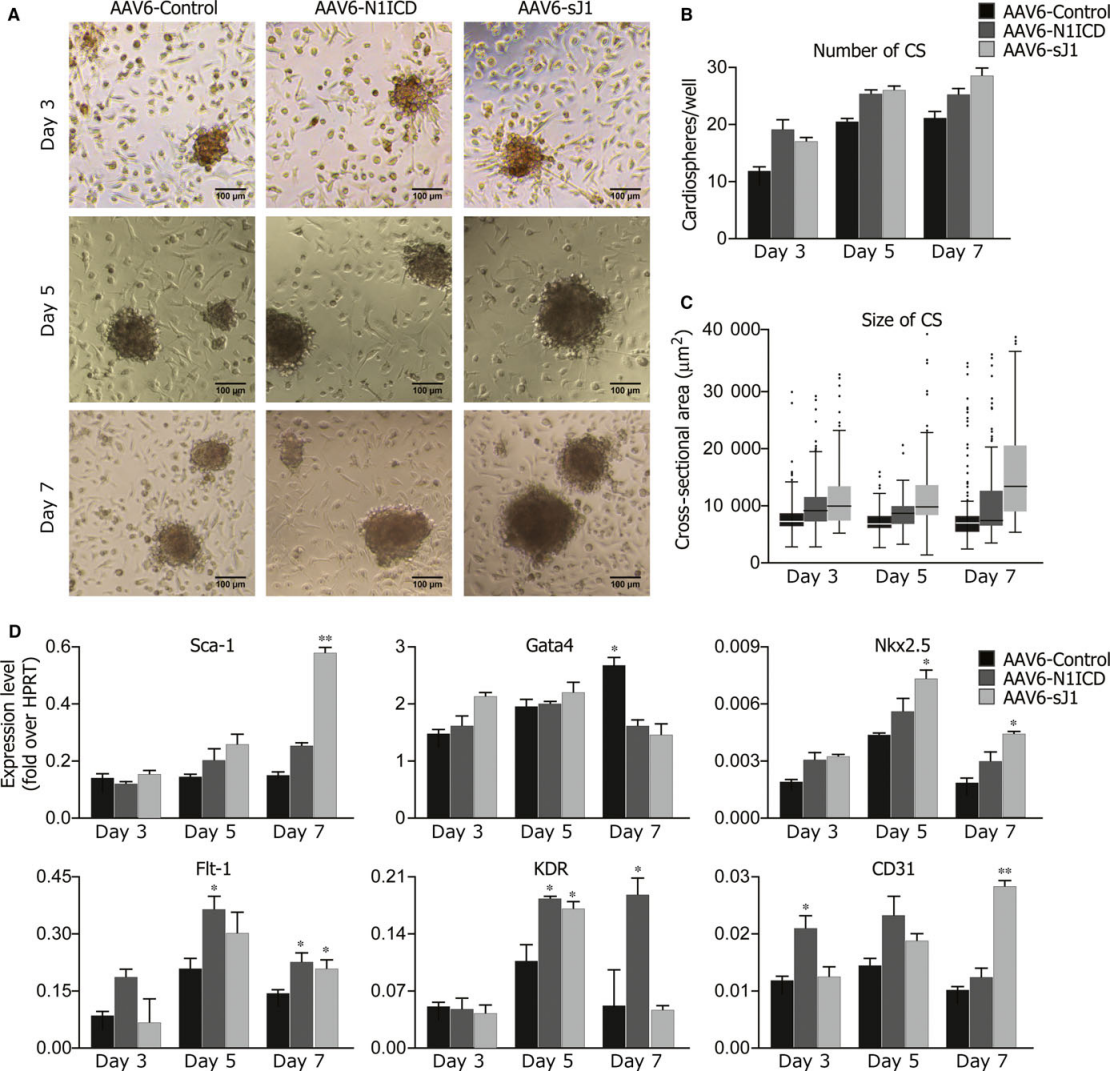
Stimulation of the Notch pathway through AAV6‐N1ICD or AAV6‐sJ1 transduction enhances cardiosphere growth and induces a cardiovascular differentiation programme. Cardiosphere‐forming cells were transduced contextually to plating with either AAV6‐MCS, ‐N1ICD or ‐sJ1 vectors (m.o.i = 1 x 10^5^). A, Representative bright field images of cardiospheres, transduced either with AAV6‐MCS, ‐N1ICD or ‐sJ1, after 3, 5 and 7 days of in vitro culture. Scale bar: 100 μm. B, Quantification of the number cardiospheres transduced either with AAV6‐MCS, ‐N1ICD or ‐sJ1, after 3, 5 and 7 days of in vitro culture. Shown are mean ± SEM of at least 3 experimental replicates. C, Distribution of the cross‐sectional areas of the cardiospheres, transduced either with AAV6‐MCS, ‐N1ICD or ‐sJ1, after 3, 5 and 7 days of in vitro culture. Shown are Tukey boxplots. D, Gene expression profile of stem‐, vasculo‐ and cardio‐related genes in cardiospheres transduced either with AAV6‐MCS, ‐N1ICD or ‐sJ1 vectors, at days 3, 5 and 7 after plating. Data are expressed to cellular HPRT mRNA levels. Shown are the mean ± SEM of at least 3 independent experiments. **P < 0.01: *P < 0.05

Both AAV6‐N1ICD and AAV6‐sJ1 markedly increased both total numbers and size of CSps at all time points (Figure 4A, B, C). The observed different kinetics between the two vectors most likely reflected a requirement for sJ1 to accumulate in the extracellular environment in order for the soluble ligand to cluster and activate the Notch pathway.^42^, ^43^ These results further support the peculiar role of the Notch pathway in CSp growth.

To elucidate the gene expression programme activated upon viral transduction, we analysed gene expression of transduced CSps at 3, 5 and 7 days of culture. Both AAV vectors triggered a gene expression programme (Figure 4D) superimposable to the one observed upon sJ1 stimulation (cf. Figure 3E), further supporting the role of Notch1 in the expansion of a pool of cells putatively committed to a cardiovascular fate.

### 3.6 miR‐199a‐3p induces Notch expression, while miR‐590‐3p enhances CSp growth and cardiovascular commitment

A previous whole‐genome, synthetic miRNA screening conducted in our laboratory identified several human miRNAs capable of inducing significant proliferation of rodent cardiomyocytes. Of interest, at least two of these miRNAs, miR‐199a‐3p and miR‐590‐3p induced cardiac regeneration after myocardial infarction using AAV‐mediated gene transfer^26^ or delivery of naked miRNA mimics.^44^ Notably, the molecular mechanism by which these miRNAs function in adult cardiomyocytes does not involve reactivation of the Notch pathway.^24^ We therefore investigated whether the most effective miRNAs in inducing neonatal and adult cardiomyocyte proliferation (hsa‐miR‐199‐3p, hsa‐miR‐590‐3p, hsa‐miR‐1825, hsa‐miR‐33b‐3p) activated the Notch pathway and played functional roles in CSps.

**Figure 5:**
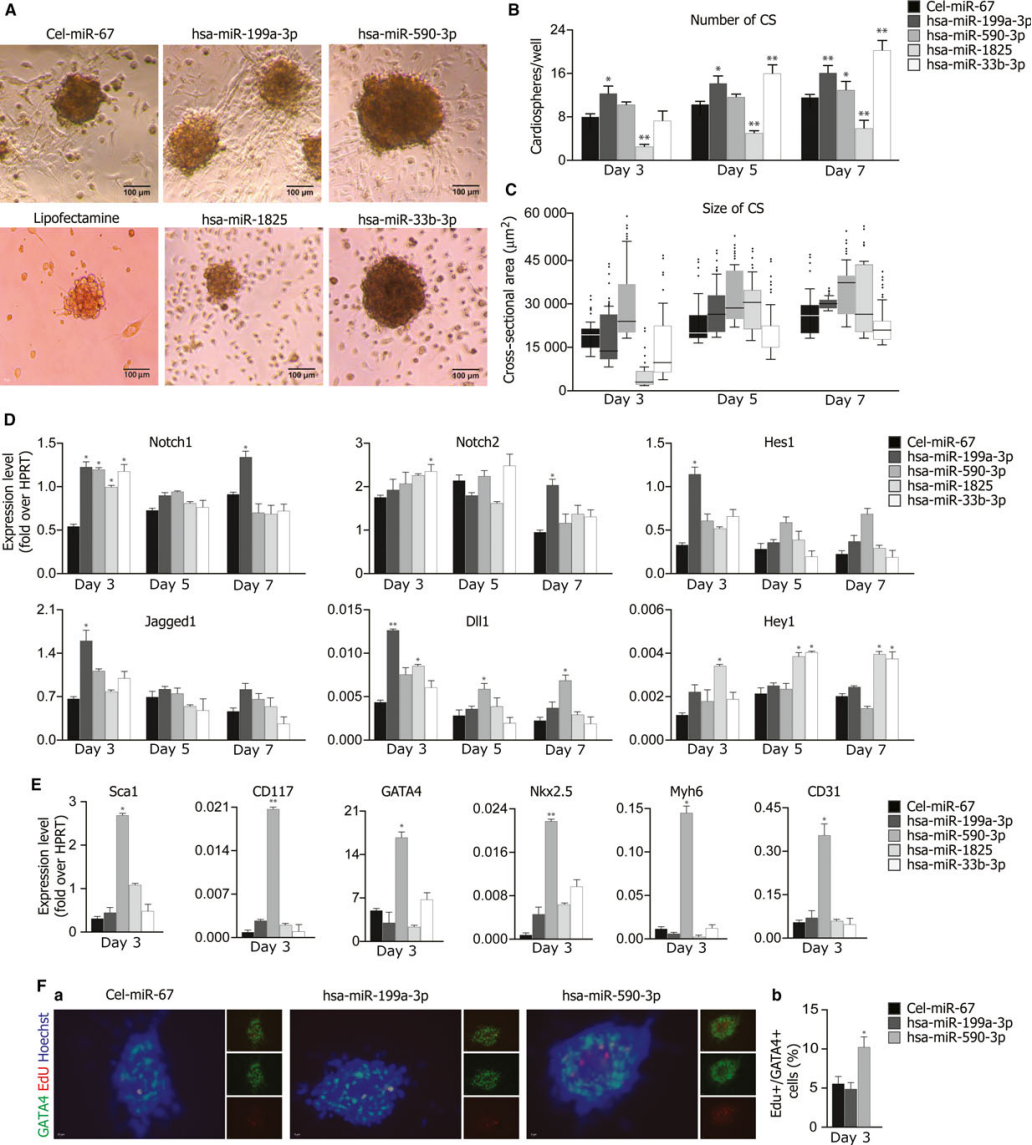
miR‐199a induces Notch signalling and enhances cardiosphere growth to a cardio‐vascular fate. Cardiosphere‐forming cells were transfected contextually to plating with either cel‐miR‐67 (control), miR‐199a‐3p, miR‐590‐3p, miR‐1825 or miR‐33b‐3p. Analyses were performed 3, 5 and 7 days after plating. A, Representative bright field images of cardiospheres, transfected with either celmiR‐67 (control), miR‐199a‐3p, miR‐590‐3p, miR‐1825 or miR‐33b‐3p, after 3, 5 and 7 days of in vitro culture. Scale bar: 100 μm. B, Quantification of the number of cardiospheres formed upon miRNA transfection, 3, 5 and 7 days of in vitro culture. Shown are mean ± SEM of at least 3 experimental replicates. **P < 0.01: *P < 0.05. C, Distribution of the cross‐sectional areas of the cardiospheres, transfected with either cel‐miR‐67 (control), miR‐199a‐3p, miR‐590‐3p, miR‐1825 or miR‐33b‐3p, after 3, 5 and 7 days of in vitro culture. Shown are Tukey boxplots. D, Transcription levels of Notch receptors, ligands and the indicated target genes in cardiospheres analysed at day 3, 5 and 7 of culture, following transfection of either cel‐miR‐67 (control), miR‐199a‐3p, miR‐590‐3p, miR‐1825 or miR‐33b‐3p. Data are expressed to cellular HPRT mRNA levels. Shown are the mean ± SEM of at least 3 independent experiments. **P < 0.01: *P < 0.05. E, gene expression analysis of stemness and cardio‐vascular markers in cardiospheres analysed after 3 days of culture, following transfection of either cel‐miR‐67 (control), miR‐199a‐3p, miR‐590‐3p, miR‐1825 or miR‐33b‐3p. Data are expressed to cellular HPRT mRNA levels. Shown are the mean ± SEM of at least 3 independent experiments. **P < 0.01: *P < 0.05. F, (a) Representative images of cardiospheres analysed after 3 days of in vitro culture, following transfection of either celmiR‐67 (control), miR‐199a‐3p, miR‐590‐3p. Gata4 (green), EdU (red), nuclei (blue). Bars: 100 μm. (b) Quantification of Gata4+ and EdU+ cells upon cel‐miR‐67 (control), miR‐199a‐3p and miR‐590‐3p transfection. Shown are mean ± SEM of at least 3 experimental replicates. **P < 0.01: *P < 0.05

CSp‐forming cells were transfected with the miRNAs contextually to plating and analyses were performed after 3, 5 and 7 days of culture. MiR‐199a‐3p, miR‐590‐3p and miR‐33b‐3p triggered a significant rise in the number and size of CSps at all time points. Of note, miR‐1825 exerted a unique effect, inducing the formation of a small number of very large CSps (Figure 5A, B, C). Gene expression analysis revealed increased Notch1 transcript levels as a result of each of the four miRNAs tested, with the highest effect observed using miR‐199a‐3p. This miRNA induced gene overexpression of Notch1 (2.3 fold over control), Jagged1 (2.1 fold over control), Dll‐1 (3 fold over control) and Hes1 (4 fold over control) after day 3 of culture (Figure 5D).

To streamline a common effect of the miRNAs of interest that could explain their similar effect on cardiosphere growth, possibly involving the Notch signalling pathway, we annotated the genes known to be differentially involved in the regulation of the Notch pathway (Gene Ontology database‐GO:0007219), in particular the negative regulators (GO:0045746), and compared them with the RNAseq data available in our laboratory, obtained by deep sequencing of RNA from rodent cardiac cells after treatment with miR‐590‐3p, miR‐199a‐3p, miR‐33b and miR‐1825 (unpublished data). We focused our attention on Neurl1a and Dner, a ligand‐specific ubiquitin ligase and an atypical Notch ligand, respectively, both involved to a various extent in the negative modulation of Notch signalling in various cellular contexts [for a review see Refs. 28, 50, 51, 52, 53]. This choice supported by the cosideration that the biological effect of each of the four miRNAs is pleiotropic, and thus the individual molecular route of action involves targeting of several different players in the same pathway(s). Figure S4 shows the bioinformatic target predictions (panels A and B) as a support of the clear downregulation of the Neurl1a and Dner transcripts in cardiospheres transfected with miR‐199a‐3p, miR‐590‐3p, miR‐1825 and miR‐33b‐3p mimics after day 3 of culture (panel C). These results support the conclusion that the most effective miRNAs in inducing cardiomyocyte proliferation exert a pro‐proliferative effect in CSps, broadly interfering with the Notch pathway regulation.

We then investigated the potential of miRNAs to induce a cardio‐specific differentiation programme. Three days after transfection, miR‐590‐3p strongly induced both early (Gata4 and Nkx2.5) and late cardiac markers (Myh6), along with the vascular marker CD31. Interestingly, miR‐590‐3p upregulated both Sca1 and CD117 transcription, suggesting a possible role for this miRNA as a positive regulator of the expansion of the pool of proliferating progenitors, which likely accounted for the observed increase in CSp size (Figure 5E).

To further characterize the effects of miR‐590 on CSps, we investigated cell proliferation. Interestingly, miR‐590‐3p transfection was associated with a two‐fold increase in EdU+/Gata4+ cells within CSps at day 3 of culture, which correlated with the observed upregulation of GATA4 transcripts (Figure 5F, panels a and b).

Collectively, these data support the notion that different miRNAs play distinct roles in CSp formation as an in vitro model of early cardiac development. MiR‐199a‐3p acts as a potent stimulus for the activation of the Notch pathway at early time points, whereas it loses its effect at later stages of CSp differentiation.^8^, ^18^, ^33^ On the other hand, miR‐590‐3p specifically promotes a cardiovascular differentiation programme, leading to the expansion of a pool of Sca1+ or CD117+ cells, and expression of cardiovascular differentiation markers.

## 4 DISCUSSION

The mammalian heart is unable to compensate for a massive loss of cardiomyocytes after injury. This is due to a dramatic decrease in the proliferative capability of mature cardiomyocytes, compared to neonatal conditions and to the poor regenerative potential of CPCs. Several approaches are under investigation to induce cardiomyocyte proliferation or progenitor activation and expansion. One of these approaches aims at expanding autologous progenitors cells from minimally invasive cardiac biopsies for cell therapy in patients after myocardial infarction (cf. CADUCEUS phase I trial Clinicaltrials.gov‐NCT00893360). This strategy was inspired by a seminal study by E. Messina and co‐workers,^19^ which established the experimental technique for in vitro growth of progenitor cells isolated from myocardial biopsies. The so‐called CSps and CSp‐derived cells produced using this technique are valuable tools for studying cardiac progenitor cell biology.

Here we have exploited the CSp model to investigate the role of the Notch pathway in the cardiovascular differentiation programme. Previous gene expression studies have shown a phenotypic heterogeneity of the cellular components of CSps.^45^, ^46^ Consistent with these findings, we detected Sca‐1 positive cells, along with cells that were more committed towards vasculo‐endothelial or cardiac fate specification. Previous evidence from our group and other laboratories have demonstrated a peculiar role of the Notch pathway in the expansion of the undifferentiated precursor cell pool in the heart and in the regulation of neonatal cardiomyocyte proliferation both in vitro and in vivo.^10^, ^12^, ^16^, ^29^, ^30^ Given the poorly differentiated molecular signature of the cell components of CSps, we wondered whether Notch signalling was involved in the regulation of CSp growth. Interestingly, we found a peculiar spatial distribution of different key molecules in the Notch pathway: immunostaining for Notch 1 and 2 receptors revealed a patchy distribution, whereas Hes1 expression was localized in the deep core of the spheres. Conversely, the ligands Jagged1 and Delta‐like1 were expressed by cells located in the outer layer of the spheres. We then either stimulated the Notch pathway with the soluble ligand Jagged1 (sJ1) or blocked its activation by inhibiting γ‐secretase processing with DAPT. The number and size of CSps was significantly increased after sJ1 treatment, with an overall upregulation of genes involved in cardiovascular development. DAPT, on the contrary, completely reversed this effect. We then decided to validate these results using a gene transfer approach involving AAV‐mediated transduction of CSp‐forming cells with either sJ1 or the activated intracellular domain of Notch1 (N1‐ICD). In both cases, we detected a net increase in both numbers and size of transduced CSps, which was associated with activation of a peculiar genetic programme promoting cardiovascular differentiation.

Multiple evidence over the last few years has shown a pivotal role of the microRNA network in the control of most biological functions of heart cells.^47^, ^48^ In particular, a previous whole‐genome, synthetic miRNA screening conducted in our laboratory revealed several human miRNAs capable of inducing significant proliferation of neonatal rat and mouse CMs.^26^ Among these miRNAs, miR‐199a‐3p, miR‐590‐3p and miR‐1825, have been shown to be involved in the regulation of embryonic cell proliferation^49^ and were capable to induce cardiac regeneration after myocardial infarction (MI), either when expressed using AAV‐mediated gene transfer^26^ or delivered as naked miRNA mimics.^44^ Remarkably, the molecular mechanisms by which the most effective pro‐proliferative miRNAs function in adult cardiomyocytes do not involve reactivation of the Notch pathway.^24^ We therefore wondered whether they could play a functional role in CSps, acting through multiple, specific pathways. All the miRNAs of interest shared a common effect on CSps, triggering a significant increase in their number and size. Gene expression analysis revealed increased levels of genes of the Notch pathway at the earliest time points of in vitro culture, as a result of each of the four miRNAs tested, suggesting this pathway as a possible target of either direct or indirect regulation by these miRNAs. While the overall mechanism of action of each miRNA is likely different, common pathways are likely to exist. Among Notch signalling negative regulators (GO:0045746), we found several different genes predicted to be differentially downregulated by each of the miRNAs of interest. In particular, we validated as possible targets Neurl1a, a ligand‐specific ubiquitin ligase^50^, ^51^ and Dner, coding for a atypical Notch ligand.^28^, ^52^, ^53^ In both cases, a net downregulation of the transcripts for these genes was observed after expression of all the tested miRNAs.

Collectively, our data show that the Notch pathway drives the proliferation of CSp‐forming cells. Exogenous activation of this pathway magnifies these effects and causes a shift of the gene expression profile towards genes involved in cardiovascular differentiation.

## DISCLOSURES

None.
