# Supplementary figures and images for: Notch pathway activation enhances cardiosphere in vitro expansion
Source: J Cell Mol Med. 2018 Aug 23;22(11):5583–95. doi: 10.1111/jcmm.13832 (PMC6201224; doi:10.1111/jcmm.13832)

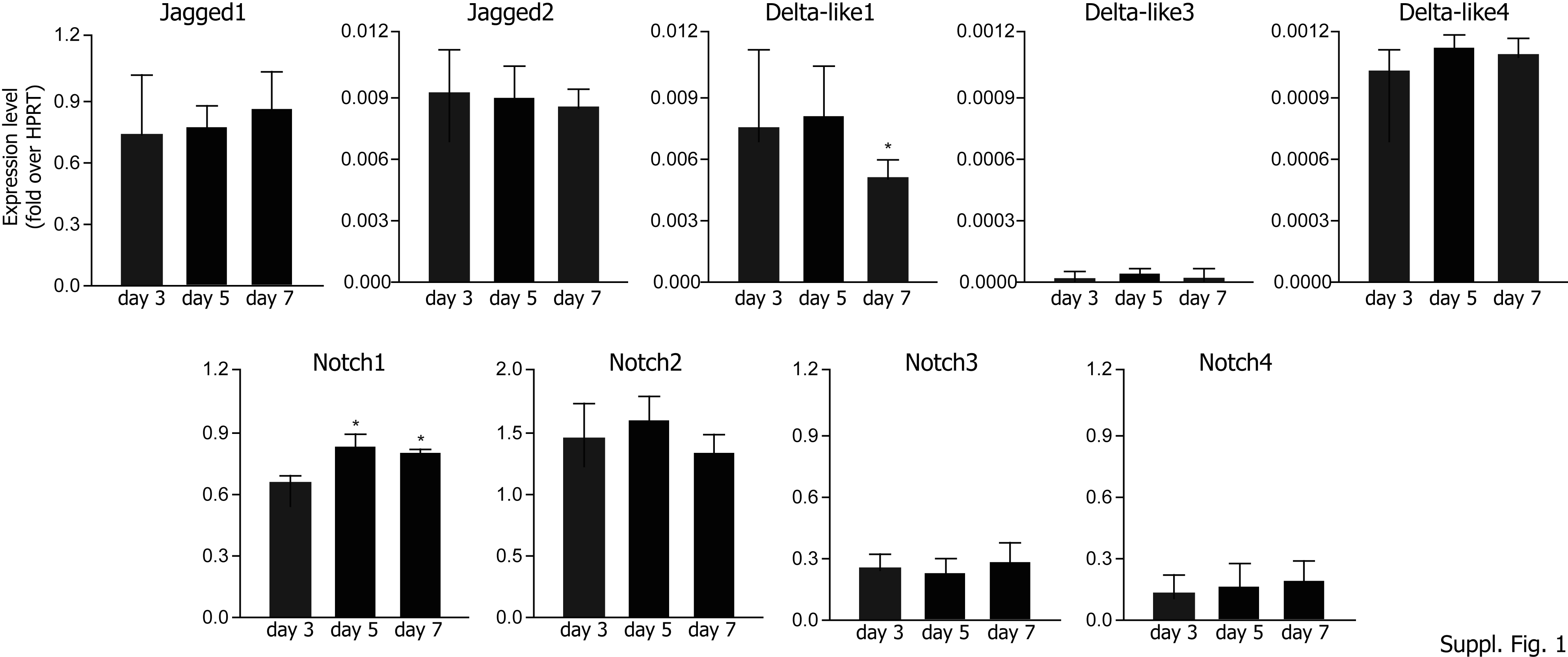

Supplement: Supplementary file 1 [file JCMM-22-5583-s001.tif]

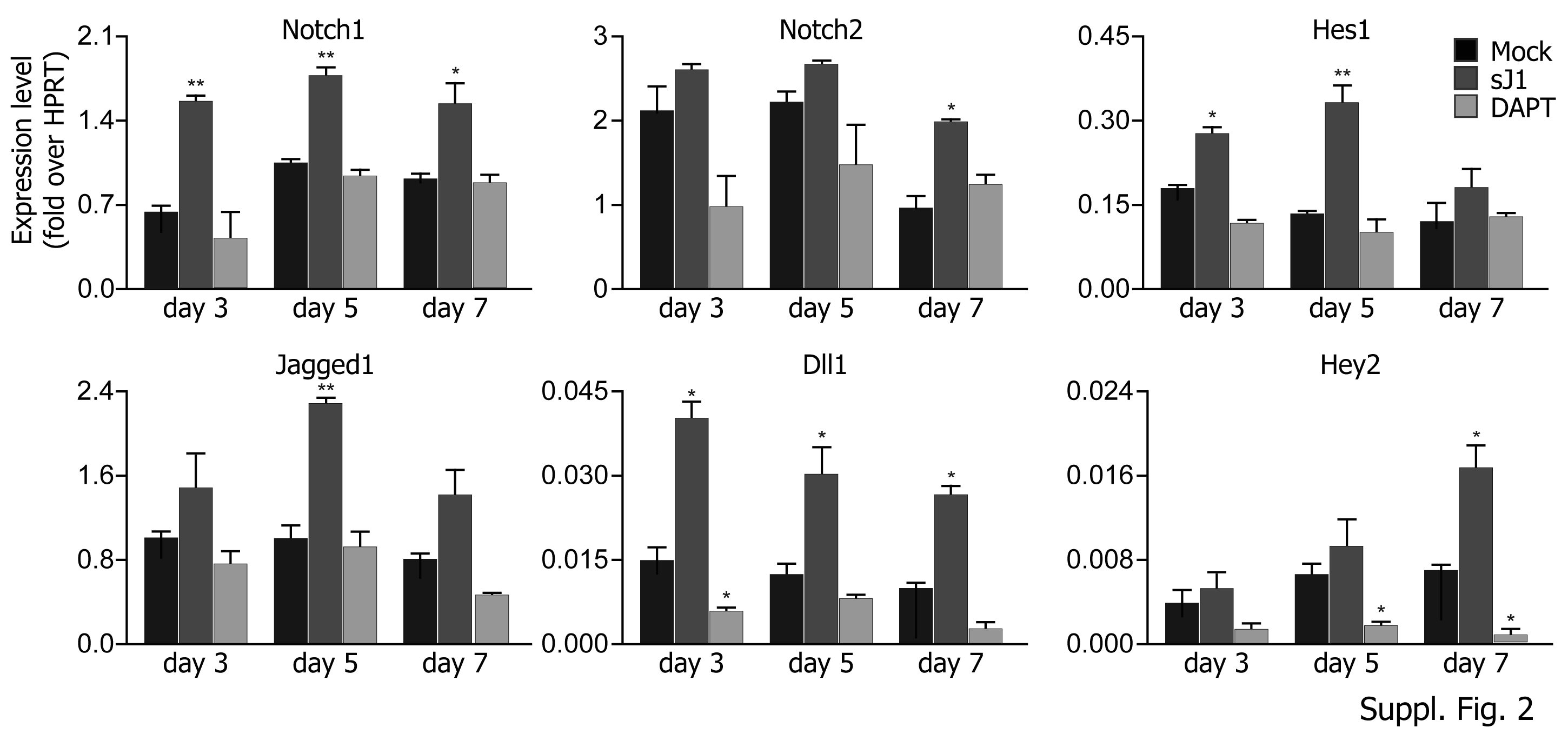

Supplement: Supplementary file 2 [file JCMM-22-5583-s002.tif]

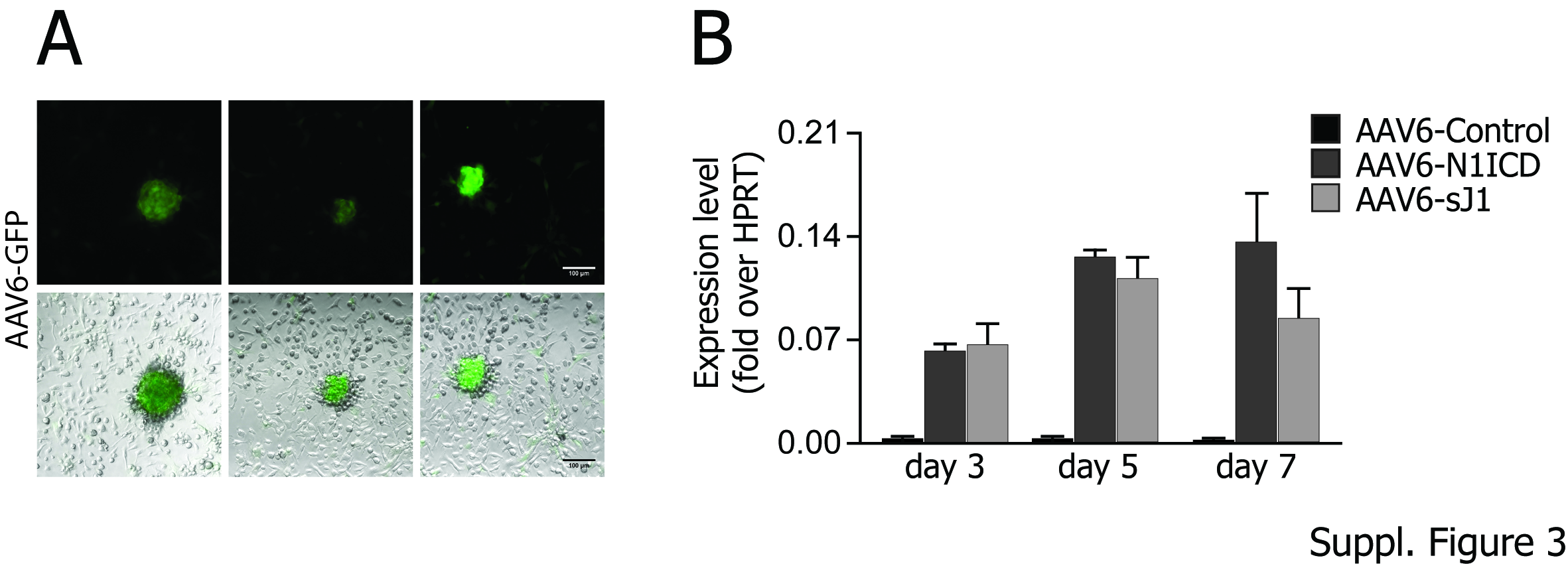

Supplement: Supplementary file 3 [file JCMM-22-5583-s003.tif]

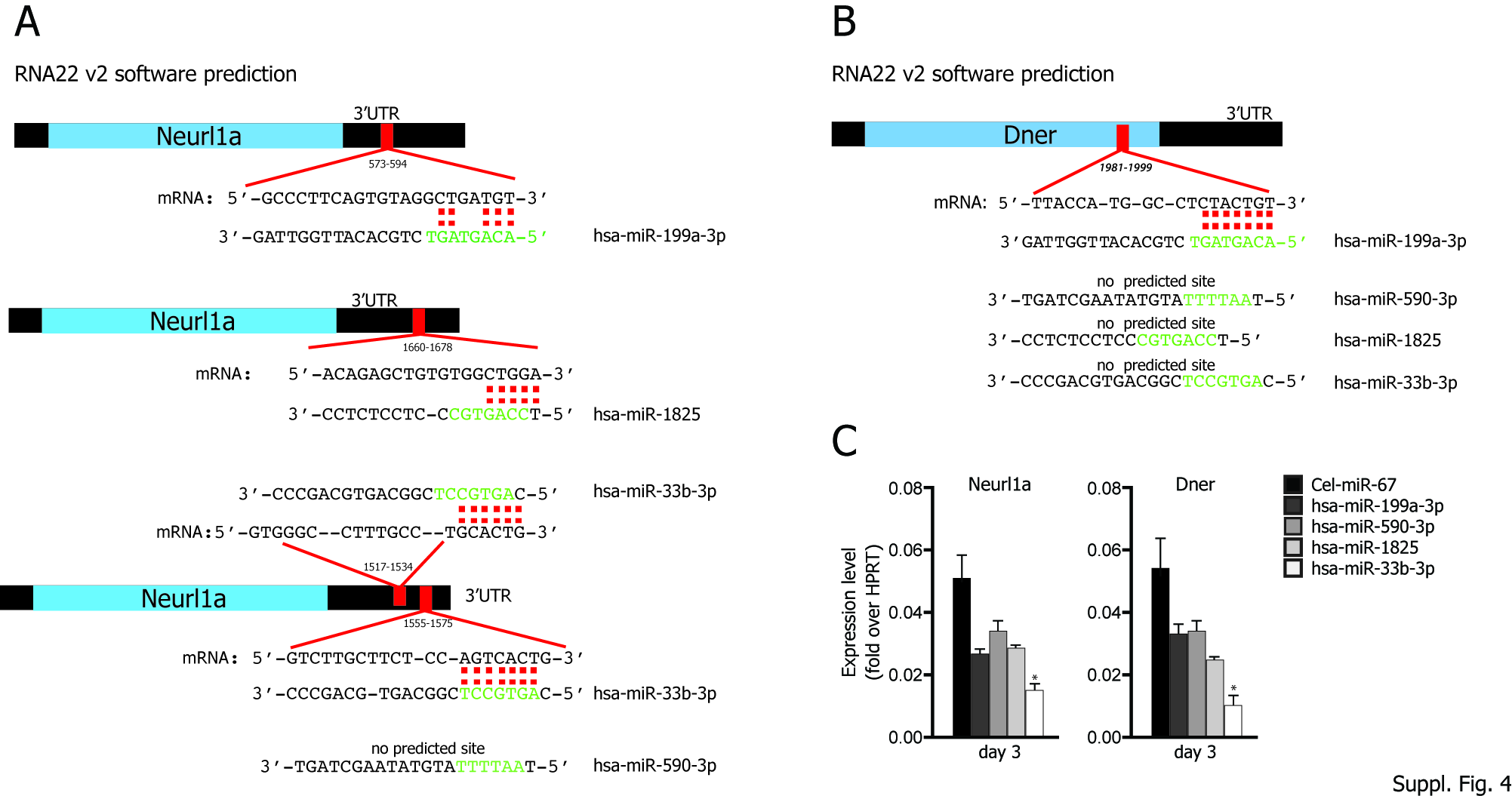

Supplement: Supplementary file 4 [file JCMM-22-5583-s004.tif]
